# The value of C-reactive protein as an independent prognostic indicator for disease-specific survival in patients with soft tissue sarcoma: A meta-analysis

**DOI:** 10.1371/journal.pone.0219215

**Published:** 2019-07-01

**Authors:** Xiaolin Wang, Song Liu, Xiaoli Zhao, Erhu Fang, Xiang Zhao

**Affiliations:** 1 Department of Pediatric Surgery, Tongji Hospital, Tongji Medical College, Huazhong University of Science and Technology, Wuhan, Hubei Province, P. R. China; 2 Department of Pediatric, Tongji Hospital, Tongji Medical College, Huazhong University of Science and Technology, Wuhan, Hubei Province, P. R. China; Duke University, UNITED STATES

## Abstract

**Backgrounds:**

Serum C-reactive protein (CRP) level has been shown to be a predictor of survival for multiple cancer types. The aim of this study was to evaluate whether pretreatment serum CRP level could serve as a reliable independent prognostic indicator for survival in patients with soft tissue sarcoma (STS).

**Methods:**

A detailed literature search was conducted in Medline, Embase and Cochrane for relevant research publications written in English. Patients’ clinical characteristics, outcomes of disease-specific survival (DSS) and disease/recurrence free survival (DFS/RFS) were extracted. Only the results of multivariate survival analysis were recruited in our analysis. Pooled hazard ratios (HRs) and corresponding 95% confidence intervals (CIs) were calculated to evaluate the prognostic role of CRP. This study was registered on PROPERO and the registration number is CRD42018104802.

**Results:**

Nine articles containing 1655 patients were identified as eligible studies. The random effects model showed that elevated CRP level was significantly correlated with poor DSS (HR = 2.08; 95% CI: 1.33–3.24; p < 0.001). After excluding the heterogeneous study, the fixed effects model showed that elevated CRP level was firmly correlated with poor DSS (HR = 2.36; 95% CI: 1.84–3.03; *p* < 0.001). The fixed effects model revealed that elevated CRP level was significantly correlated with poor DFS (HR = 1.78; 95% CI: 1.39–2.30; *p* < 0.001) among studies have more than 100 samples.

**Conclusion:**

The results of this meta-analysis suggest that elevated pretreatment serum CRP level could serve as an independent risk factor for poor DSS and DFS/RFS in STS patents.

## Introduction

Soft tissue sarcoma (STS) represents a heterogeneous group of tumors that arise predominantly from the embryonic mesoderm, with diverse subtypes and varying degrees of aggressiveness [1]. Traditional prognostic factors such as histologic grade, histological subtype, tumor size, tumor depth and anatomical location have been used to conduct risk assessment and make decisions regarding surgical strategy, adjuvant management and surveillance [2]. However, the overall 5-year survival rate in STS patients of all stages accounts for only 50%-60% [1], and about 50% of patients with adequate local control develop distant metastases and ultimately die from their disease [2]. Thus, further improvement of prognostic classification is warranted for better treatment options and surveillance, preferably using readily available clinical parameters that show better predictive power.

It has become increasingly accepted that certain systemic inflammatory responses may play important roles in cancer progression and metastasis [3–5]. Tumor associated inflammatory responses may lead to alteration in cancer cell biology and activation of stromal cells in tumor microenvironment by upregulation of cytokines and inflammatory mediators, inhibition of apoptosis, induction of angiogenesis, stimulation of DNA damage and immunosuppression and remodeling of the extracellular matrix, hence promoting tumor growth and metastasis [4, 5].

C-reactive protein (CRP) is a non-specific blood based marker of acute-phase inflammatory response, and it is a readily accessible but cheap laboratory parameter widely used in clinical routine. Serum CRP levels have been shown to be elevated in patients with multiple types of cancers, and, in particular, elevated serum CRP levels have been associated with poor survival [6–12]. Recently, a study revealed that high serum CRP level was significantly associated with PD-L1 (programmed death-ligand 1) positivity in patients with non-small cell lung cancer [13]. It suggests a potential role for CRP serve as an indicator for immune checkpoint blockade therapy with anti-PD-1 (programmed death 1) antibodies.

The inflammatory status may also be a prognostic factor for STS. Recently, several studies have been conducted to assess the prognostic significance of inflammatory markers, including CRP, on the survival of STS patients [14–22]. In this study, we conducted a meta-analysis and combined the results of relevant studies to evaluate whether pretreatment serum CRP level could serve as a reliable independent prognostic indicator for cancer-specific survival of STS patients.

## Methods

### Search strategy

From January 1990 to March 2019, the relevant literature from the Medline, Embase and Cochrane databases was systematically screened. The latest search was performed on March 10, 2019. The keywords “C-reactive protein” and “sarcoma” were used for the preliminary search. At the same time, relevant studies were also identified by a manual search of references of initially identified articles. Nonhuman study or non-English articles were excluded. Two investigators reviewed the titles and abstracts identified in the search.

This study was registered on PROPERO and the registration number is CRD42018104802.

### Study inclusion/exclusion criteria

Studies were considered eligible if they met all of the following inclusion criteria: (1) studies conducted on patients with soft tissue sarcoma; (2) studies investigated the relationship between pretreatment serum CRP levels and disease-specific survival (DFS) or disease/recurrence free survival (DFS/RFS). (3) studies provided data about hazard ratios (HRs) along with their 95% confidence interval (CIs) by multivariate survival analysis.(4) case-control studies, cohort studies and RCTs. Studies were excluded based on any of the following exclusion criteria: (1) literature published as case reports, letters, editorials, abstracts, reviews or expert opinions; (2) studies based not on human STS patients; (3) articles provided only outcomes of univariate survival analysis; (4) studies focus on sarcoma of bone or any other kind of non-soft tissue sarcoma. When the same patient population was involved in two or more studies, only the last or complete study was chosen.

### Data extraction

Eligible publications were reviewed independently by two investigators. The data extraction was performed by two investigators. Disagreements were resolved by consensus between the reviewers. A standardized data collection form defined previously with the following items: first author, year of publication, period of enrollment, study design, country of origin, sample size, histology type, metastasis case numbers, tumor size, follow-up period, CRP level cut-off methods and cut-off values, and HRs estimates with corresponding 95% CIs. For each study, we extracted the risk estimates that were adjusted for the greatest number of potential confounders. DSS was the primary outcome for this meta-analysis. DFS/RFS were the secondary outcome.

### Assessment of quality

Two reviewers independently assessed the risk of bias for each study. The Newcastle-Ottawa Quality Assessment Scale (NOS) was applied to assess qualities of cohort studies. A study with NOS > 5 was regarded as a high-quality study [23].

### Statistical analysis

Statistical analysis was carried out using the STATA statistical software package version 12.0 (Stata Corporation, College Station, TX). Combined HRs and Forrest plots were used to estimate the predictive role of CRP levels in STS patients. The Cochrane Q test (P<0.05 indicated a high level of heterogeneity) and I^2^ (values of 25%, 50% and 75% corresponding to low, moderate, and high degrees of heterogeneity, respectively) was used to evaluate the heterogeneity between eligible studies. When homogeneity was good, a fixed-effect model was used; When heterogeneity was high, a random-effect model was used [24]. An observed HR > 1 indicated worse outcome for higher CRP levels. Begg’s test and Egger’s test on asymmetry of funnel plot were performed to test any existing publication bias. If evidence of publication bias was found, trim and fill method was adopted to check and revise the combined HRs [25]. Meta-regression analyses and subgroup analyses were performed to investigate the sources of heterogeneity [26]. Sensitivity analysis was also conducted to assess the influence of each individual study on the strength and stability of the combined HRs. All statistical tests were two-tailed and *p* < 0.05 was considered statistically significant.

## Results

### Search results

**Fig 1:**
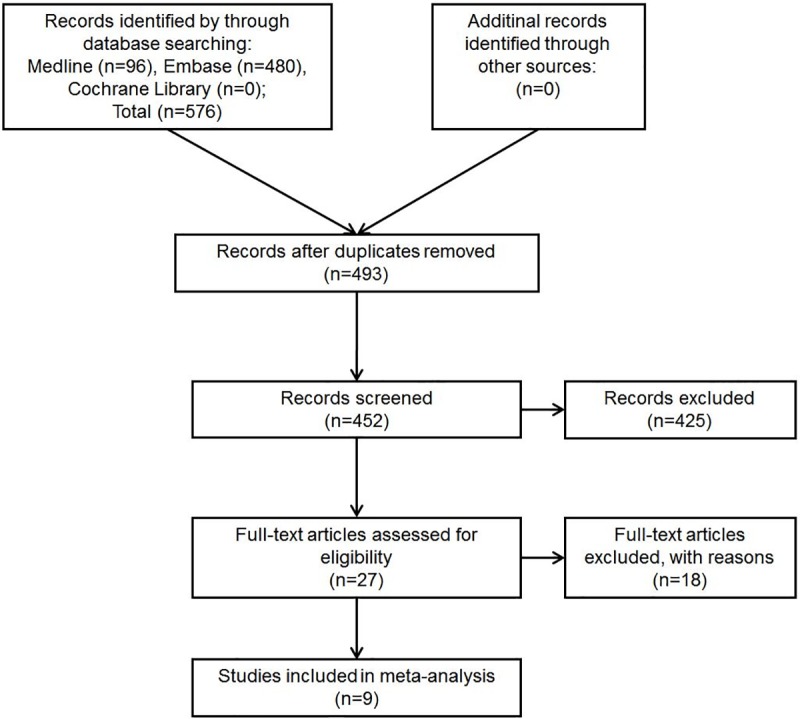
Flowchart presenting the steps of records search and selection.

Our literature search flow chart is shown in Fig 1. Five hundred and seventy-six records in total were found in the initial search of the three data bases, and 83 duplicate articles were deleted after duplicate checking. Nonhuman researches and non-English articles were also removed. Four hundred and fifty-two records were left for titles and abstracts screening. After two evaluators’ discussion, 27 articles were regarded as potentially relevant articles for full text review. Seven articles were removed as they investigated sarcoma of bone or involved sarcoma of bone [27–33]. Two articles were removed as they involved the same group of STS patients [34, 35]. Seven articles were removed due to lack of survival data for CRP [36–42]. Two articles were excluded as they provided only OS data [16, 43]. Finally, 9 eligible studies with 1655 patients were included in this meta-analysis [14, 15, 17–22, 44].

### The characteristics of the included studies

Nine cohort studies from 2012 to 2017 evaluating the relationship between CRP levels and prognosis of STS were included, with 1655 patients. The main features of the 9 included studies were summarized in Table 1. In short, 5 studies were conducted in Europe (1 in UK, 1 in Denmark and 3 in Austria), 3 in Asia (2 in Japan and 1 in Korea), while the other one in U.S.A. The sample size ranged from 47 to 403, while the follow-up time ranged from 28.4 months to 5.7 years. Two of them is prospective cohort study [20, 22], while all the other 7 were retrospective studies. Four studies focused on STS patients with localized primary tumor at initial presentation [15, 18, 20], another 4 studies focused on STS patients with no restriction on metastasis or not [17, 19, 44], and only 1 study focused on STS patients with metastasis at initial presentation [21]. Only 1 of the 9 studies declared that patients with medical conditions known to affect systemic inflammation status were excluded [18].

**Table 1:** Basic characteristics of the included studies.

Study	Country	Study design	Study period	Sample size (n)	Histology types	Metastasis case (n)	Follow-up	Tumor sizes (cm)	CRP (mg/dL)
**Nakamura T** **2012**	Japan	Rs	2003–2009	102	well-differentiated liposarcoma (n = 22), myxofibrosarcoma (n = 13), leiomyosarcoma (n = 12), malignant fibrous histiocytoma (n = 11), myxoid liposarcoma (n = 9), malignant peripheral nerve sheath tumor (n = 6), extraskeletal chondrosarcoma (n = 5), synovial sarcoma (n = 5), dermatofibrosarcoma protuberances (n = 5),other tumors (n = 14).	0	mean: 35 m range: 7–87 m	mean: 8.4 range: 0.4–20.4	mean: 2.3^a^ range: 0.4–20.4^a^
**Nakamura T** **2013**	UK	Rs	2003–2010	332	malignant fibrous histiocytoma (n = 119), liposarcoma (n = 48), myxofibrosarcoma (n = 43), leiomyosarcoma (n = 33), synovial sarcoma (n = 23), fibrosarcoma (n = 11), malignant peripheral nerve sheath tumor (n = 10).	0	mean: 28.4 m range: 1–101 m	mean: 10.6 range: 1–30	mean: 7.5^a^ range: 1.1–34.2^a^
**Szkandera J** **2013**	Austria	Rs	1998–2010	304	myxofibrosarcoma (n = 91), liposarcoma (n = 75), leiomyosarcoma (n = 37), synovial sarcoma (n = 26), malignant peripheral nerve sheath tumour (n = 13), other tumors (n = 62).	12	mean: 36 m range: 0–162 m	mean: 9.5±6.7 range: 1–47	median: 0.33 IQR: 0.1–1.15
**Choi ES2014**	Korea	Rs	1999–2011	162	liposarcoma (n = 48), malignant fibrous histiocytoma (n = 31), synovial sarcoma (n = 15), leiomyosarcoma (n = 11).	0	mean: 46.7 m range: 6–144 m	mean: 8.0 range: 1.1–35.0	mean: 0.79 range: 0.01–17.10
**Panotopoulos J** **2015**	Austria	Rs	1994–2011	85	liposarcoma (n = 85).	2	mean: 5.6 y range: 0.1–20.4 y	NA	median: 0.5 range: 0.01–13.2
**Maretty-K K** **2017**	Denmark	Ps	1994–2013	403	liposarcoma(n = 81), undifferentiated pleomorphic sarcoma (n = 71), leiomyosarcoma (n = 71), dermatofibrosarcoma (n = 28), synovial sarcoma (n = 24), malignant peripheral nerve sheath tumor (n = 24), other tumors (n = 104).	0	mean: 5.7 y range: 0.1–22 y	mean: 7 range: 1–40	NA
**Willegger M** **2017**	Austria	Rs	1996–2016	132	fibrosarcoma (n = 20), fibromyxosarcoma (n = 3), myxofibrosarcoma (n = 95), spindle cell sarcoma (n = 12), sclerosing epitheloid fibrosarcoma (n = 2).	13	mean: 4.3 y 95%CI: 3.3–5.1 y	NA	median: 0.7 IQR: 0.2–2.4
**Nakamura T** **2017**	Japan	Rs	2008–2013	47	undifferentiated pleomorphic sarcoma (n = 15), leiomyosarcoma (n = 7), synovial sarcomas (n = 6), alveolar soft part sarcoma (n = 5), liposarcoma (n = 4), malignant peripheral nerve sheath tumor (n = 4), other tumors (n = 6).	47	mean: 24 m range: 1.5–72.5 m	mean: 9.7 range: 4–25	mean: 2.47 range: 0.01–32.95
**Yanagisawa M** **2018**	U.S.A	Ps	2007–2015	49×2 ^b^	high grade undifferentiated pleomorphic sarcoma (n = 35), liposarcoma (n = 18), leiomyosarcoma (n = 8), other tumors (n = 37).	0	31.8 m	mean: 3.3±5.6	mean: 9.5 range: 0.7–60

Rs, retrospective study; Ps, prospective study; m, month; y, year; NA, data not available; IQR, Inter Quartile Range; CI, confidence interval; Cx, chemotherapy; Rx, radiotherapy; HPT, heavy particle therapy.

^a^ data from patients group with elevated CRP levels.

^b^ this study has two independent cohorts, 49 patients respectively.

Survival analysis outcomes of the included studies were summarized in Table 2. CRP level was analyzed as continuous variables (per 1 log mg/dL increase) in two studies [19, 44], and as dichotomous variables (lower CRP level versus higher CRP level) in the others. Seven studies provided the DSS data, 6 of them concluded that higher CRP level was statistically correlated with poor DSS [15, 17–21], and only 1 of them found no statistical association between CRP level and DSS [44]. Five articles provided the DFS/RFS data, 4 of them concluded that higher CRP level was statistically correlated with poor DFS/RFS, 1 article with 2 independent cohorts concluded that higher CRP level was not statistically correlated with poor distant-recurrence free survival (DRFS). For the DFS/RFS, 3 studies focused on both local recurrence and distant metastasis [14, 17, 19], 1 focused on local recurrence [15], the other 1 focused on distant recurrence which means distant metastasis[22].

**Table 2:** Survival analysis data of the included studies.

Study	Cut-off value	Cut-off method	Survival analysis	HR	95% CI	*P* value	Variables for multivariate analysis
**Nakamura T** **2012**	0.3 mg/dL	Clinical routine	DFS	2.78	1.19–6.25	0.017	gender, tumor grade
**Nakamura T** **2013**	1 mg/dL	Clinical routine	DSS	3.94	2.23–6.94	< 0.0001	tumor grade, tumor size, AJCC stage
			RFS	2.23	1.1–4.85	0.04	
**Szkandera J** **2013**	0.69 mg/dL	ROC	CSS	2.25	1.21–4.18	0.01	age, gender, tumor depth, tumor grade, tumor size, AJCC stage
			DFS	1.97	1.13–3.45	0.017	
**Choi ES** **2014**	0.2 mg/dL	ROC	DSS	3.18	1.21–6.40	0.019	ESR, NLR
**Panotopoulos J** **2015**	continuous variable	continuous variable	DSS	1.92	0.77–4.8	0.17	AJCC stage
**Maretty-K K** **2017**	0.3 mg/dL	Reference	DSS	1.8	1.1–3.0	0.02	age, tumor size, tumor grade, histological type, tumor depth, comorbidity
**Willegger M** **2017**	continuous variable	continuous variable	DSS	2.02	1.19–3.41	0.009	AJCC stage
			RFS	1.54	1.11–2.14	0.01	
**Nakamura T** **2017**	0.3 mg/dL	Clinical routine	DSS	1.108	1.029–1.194	0.007	age, primary surgical resection, Hemoglobin, Albumin
**Yanagisawa M** **2018**	0.5 mg/dL	Median value	DRFS	0.96 ^a^	0.84–1.10 ^a^	0.54 ^a^	Age, tumor size, histology, NLR
				1.02 ^b^	0.96–1.08 ^b^	0.49 ^b^	

ROC, ROC curve; DSS, disease specific survival; DFS, disease free survival; RFS, recurrence free survival; DRFS, distant-recurrence free survival; HR, Hazard ratio; CI, confidence interval; ESR, erythrocyte sedimentation rate; NLR, neutrophil to lymphocyte ratio; AJCC, American Joint Committee on Cancer.

^a^ data for patients group received preoperative radiotherapy

^b^ data for patients group received upfront surgery

### Relationship between CRP levels and DSS

**Fig 2:**
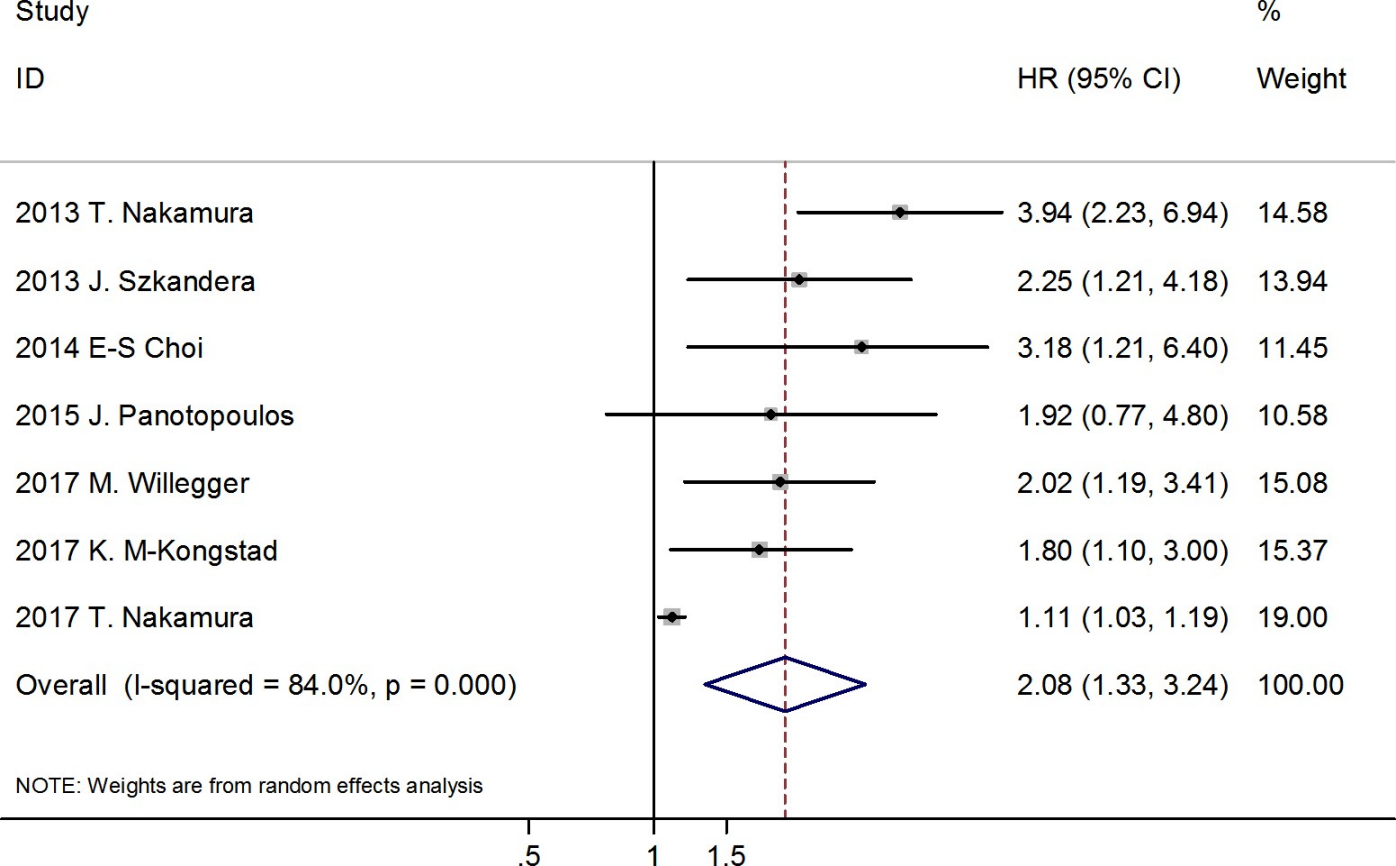
Forest plot showing the association between CRP levels and DSS of patients with soft tissue sarcoma. The summary HR and 95% CIs were shown.

Seven of the 9 included studies reported the relationship between pretreatment serum CRP levels and DSS in patients with STS [15, 17–21, 44]. The random effects model showed that higher CRP levels were significantly correlated with poor DSS (HR = 2.08; 95% CI: 1.33–3.24; *p* < 0.001), but with significant heterogeneity (I^2^ = 84.0%, *p* < 0.001) (Fig 2).

#### Sources of heterogeneity

In order to identify the sources of heterogeneity, meta-regression analysis and sensitivity analysis were conducted.

Meta-regression analysis (Table 3) revealed that the metastasis status (with or without metastasis at initial presentation) and sample sizes (n > 100 or n < 100) were responsible for the heterogeneity (R^2^ = 78.47% and R^2^ = 79.08, respectively). All of the study design (prospective or retrospective), patients’ country regions (Europe or Asia) and variable types (continuous or dichotomous) were not the sources of heterogeneity.

**Table 3:** Meta-regression analysis for the relationship between CRP levels and DSS.

Variables		*P* value	Tau^2^ value	Adj R^2^ value
**metastasis or not**	with metastasis	reference	0.034	78.47%
	without metastasis	0.034		
	mixed	0.094		
**sample sizes**	n>100	reference	0.033	79.08%
	n<100	0.028		
**study design**	prospective	reference	0.193	-23.07%
	retrospective	0.794		
**country regions**	Europe	reference	0.111	29.21%
	Asia	0.267		
**variable types**	dichotomous	reference	0.13	16.94%
	continuous	0.975		

**Fig 3:**
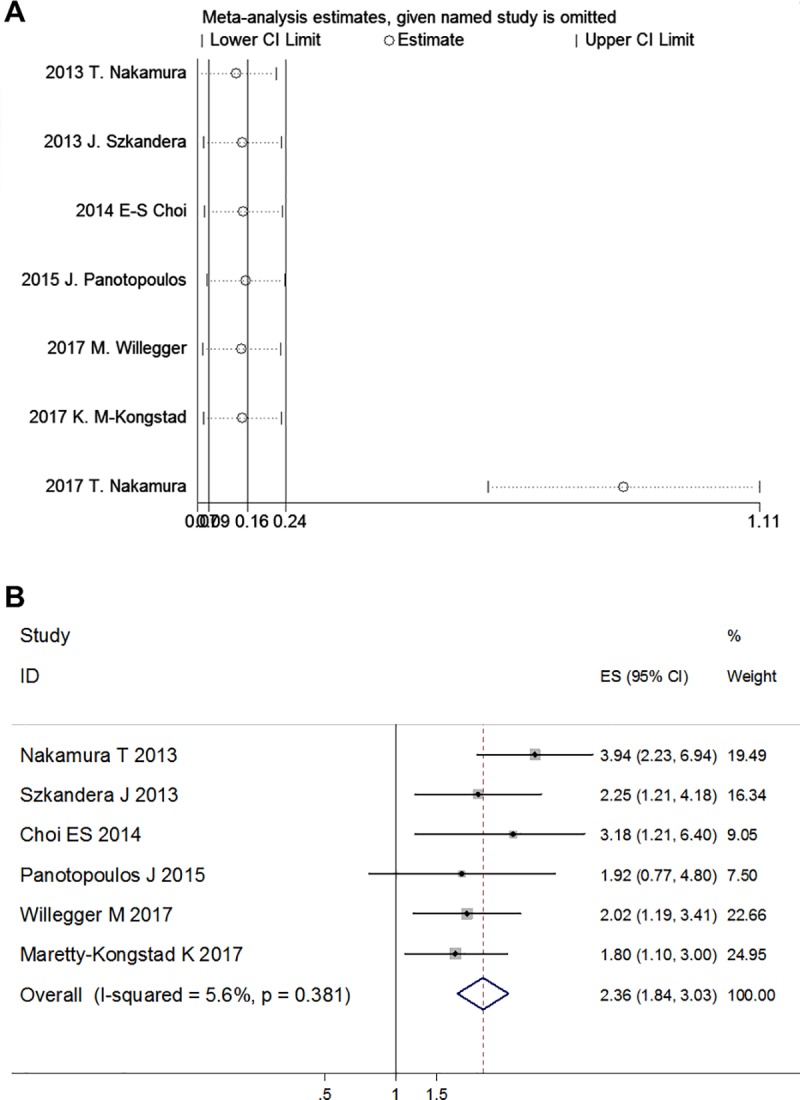
Sensitivity analysis for the relationship between CRP levels and DSS. (A) Influence of each study on the pooled HRs. (B) Forest plot after the heterogeneous study was excluded.

Sensitivity analysis conducted by excluding each one of the included studies revealed that, the study conducted by Nakamura T et al. in 2017 had significant influence on the combined HR (Fig 3A). This study have the smallest sample size (n = 47), and is the only one focus on metastatic STS patients (with metastasis at initial presentation). After excluding this study, the heterogeneity disappeared (I^2^ = 5.6%, *p* = 0.381), and the fixed effects model showed that elevated CRP level was firmly correlated with poor DSS among the remaining 5 studies (HR = 2.36; 95% CI: 1.84–3.03; *p* < 0.001) (Fig 3B).

From the above, we concluded that different metastasis status and different sample sizes were the major sources of heterogeneity. The study conducted by Nakamura T et al. in 2017 is the most heterogeneous study, which have the smallest sample size (n = 47), and is the only one focus on metastatic STS patients.

#### Subgroup analysis

We also conducted subgroup analyses based on different metastasis status and different sample sizes respectively.

**Fig 4:**
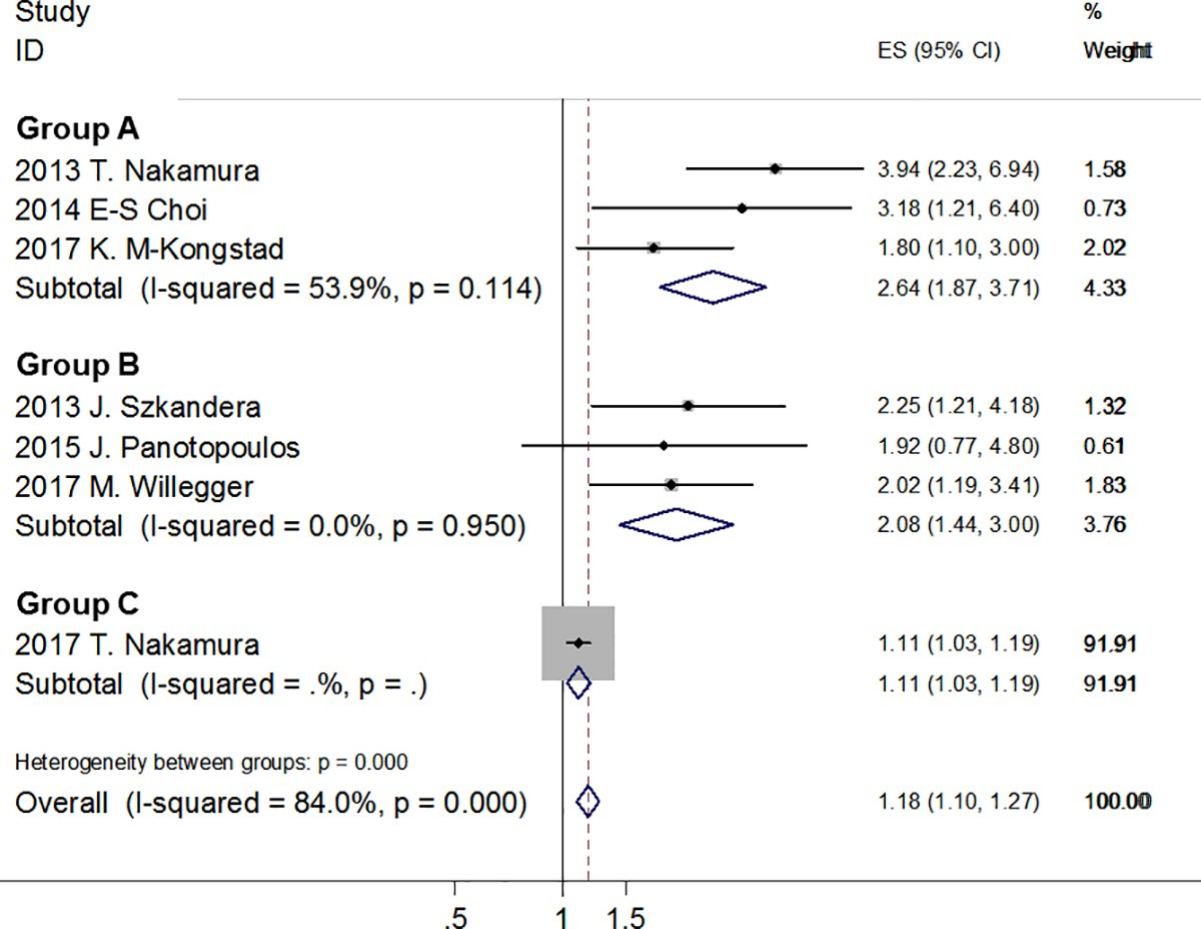
Forest plot of subgroup analysis for the relationship between CRP levels and DSS based on different metastasis status.

Based on the different metastasis status of the STS patient population, we divided the 7 studies into 3 groups: Group A, studies focus on non-metastatic STS patients (with localized primary tumor at initial presentation), which have 3 studies [15, 18, 20]; group B, studies focus on mixed STS patients (either with or without metastasis at initial presentation), which have 3 studies [17, 19, 44]; and group C, study focus on metastatic STS patients (with metastasis at initial presentation), which have only one study [21]. For non-metastatic STS patients, the random effects model showed that higher CRP levels was correlated with poor DSS (HR = 2.74; 95% CI: 1.62–4.63; *p* < 0.001), with moderate heterogeneity (I^2^ = 53.9%, *p* = 0.114); for mixed STS patients, the fixed effects model showed that higher CRP level was correlated with poor DSS (HR = 2.08; 95% CI: 1.44–3.00; *p* < 0.001), with no significant heterogeneity (I^2^ = 0.0%, *p* = 0.95); only one study–the study conducted by Nakamura T et al. in 2017 –was conducted on metastatic STS patients, which revealed that elevated CRP levels was associated with poor DSS (HR = 1.11; 95% CI: 1.03–1.19; *p* = 0.007), but with a slightly lower HR value compare with both of the other 2 groups (Fig 4).

**Fig 5:**
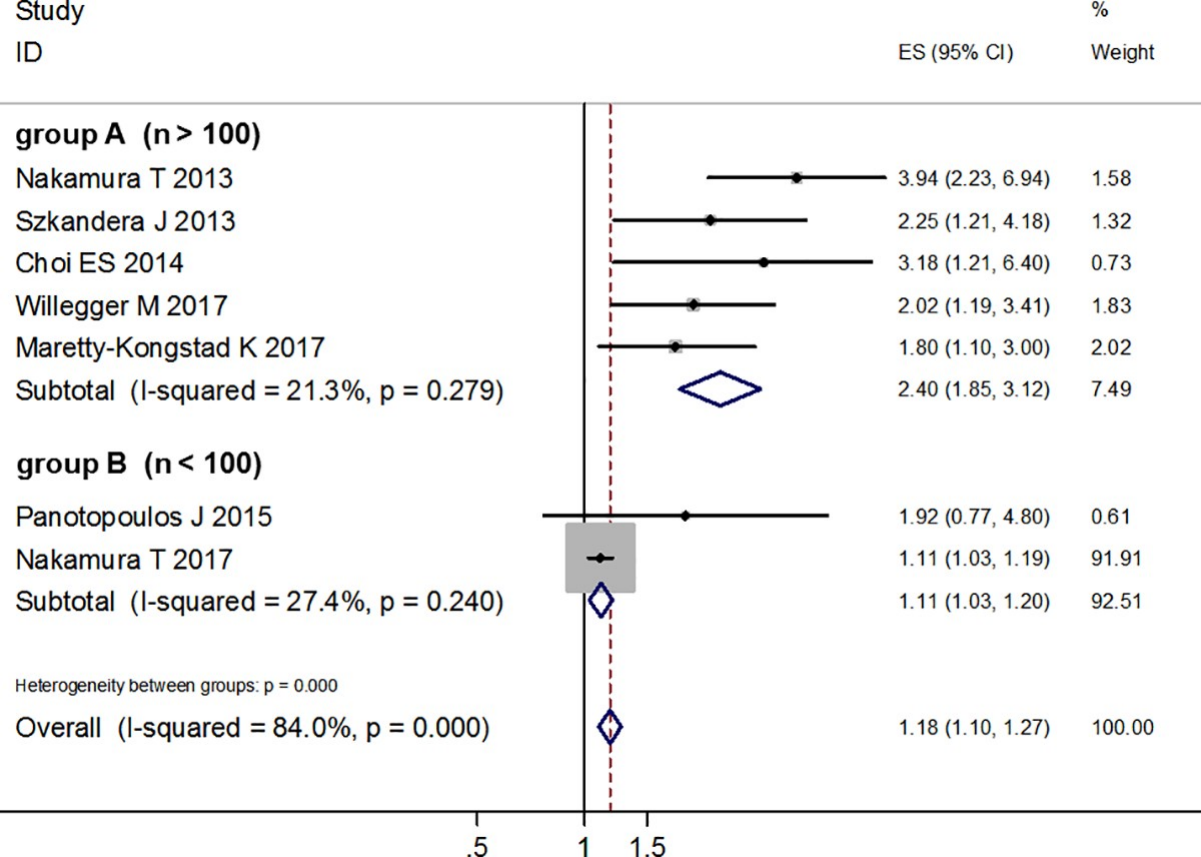
Forest plot of subgroup analysis based on different sample sizes.

Based on different sample sizes (n > 100 or n < 100), we divided the 7 studies into 2 groups: Group A, studies have more than 100 samples [15, 17–20]; group B, studies have less than 100 samples [21, 44]. There was no significant heterogeneity within each of the subgroup. For group A, the fixed effects model showed that higher CRP level was correlated with poor DSS (HR = 2.40; 95% CI: 1.85–3.12; *p* < 0.001); for group B, the fixed effects model also showed that higher CRP level was correlated with poor DSS (HR = 1.11; 95% CI: 1.03–1.20; *p* = 0.005), but the combined HR value is lower than the combined HR value of group A (HR = 1.11 vs HR = 2.40) (Fig 5).

#### Publication bias

**Fig 6:**
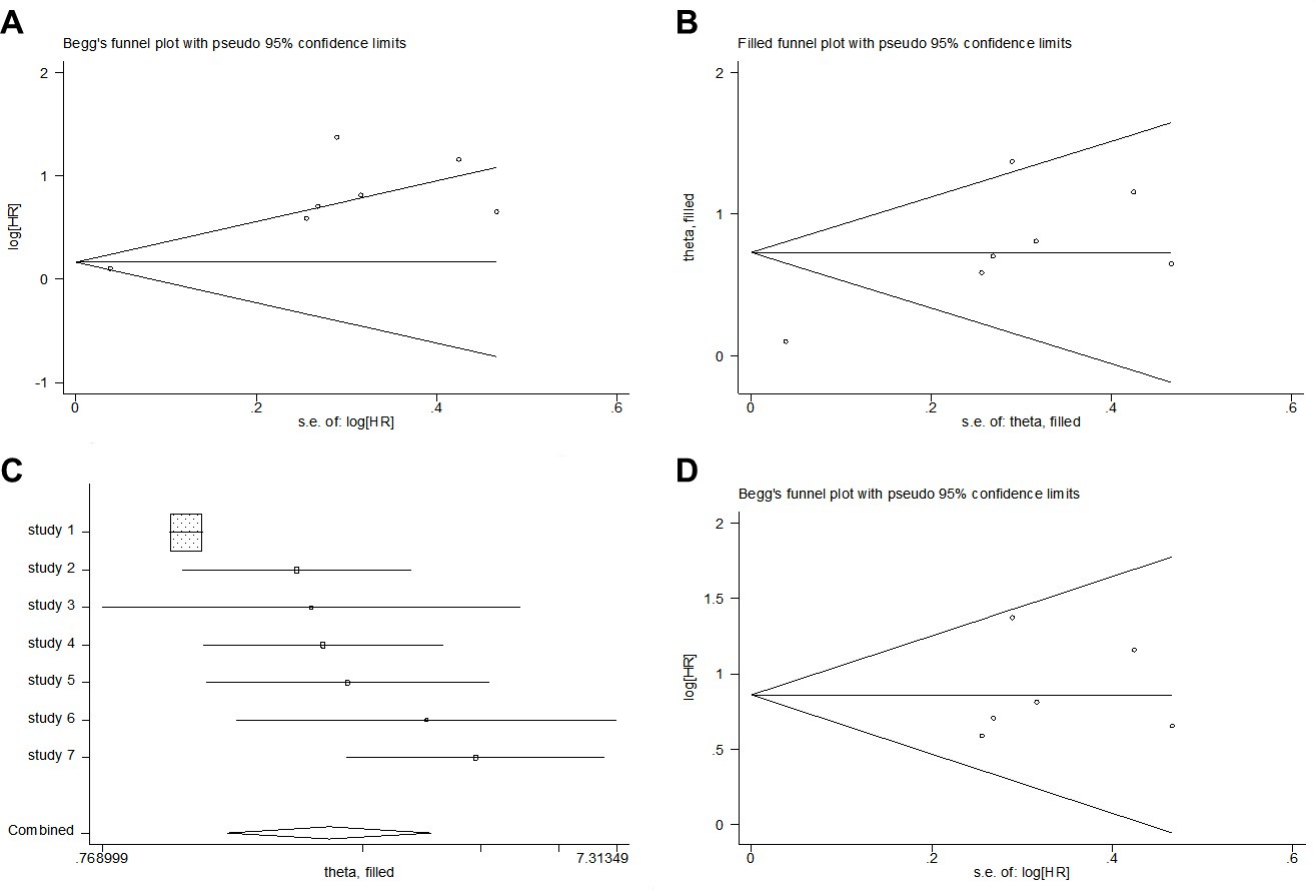
Analyses of publication bias for the relationship between CRP levels and DSS. (A) Funnel plot of all 7 studies. (B) Funnel plot after the trim and fill method was adopted. (C) Forest plot after the trim and fill method was adopted. (D) Funnel plot after the heterogeneous study was excluded.

Among all the 7 included studies, the Begg’s test showed no evidence of significant publication bias (*p* = 0.368), but the Egger’s test showed evidence of significant publication bias (*p* = 0.004) (Fig 6A). Then, the trim and fill method was adopted, but the results showed no changes between the previous and new HRs (HR = 2.08; 95% CI: 1.33–3.24; *p* < 0.001; random effects) (Fig 6B and 6C). After excluding the heterogeneous study conducted by Nakamura T et al. in 2017, the Begg’s test and Egger’s test both showed no evidence of significant publication bias (*p* = 0.452 and *p* = 0.713, respectively) for the remaining 6 studies (Fig 6D).

### Relationship between CRP levels and DFS/RFS

**Fig 7:**
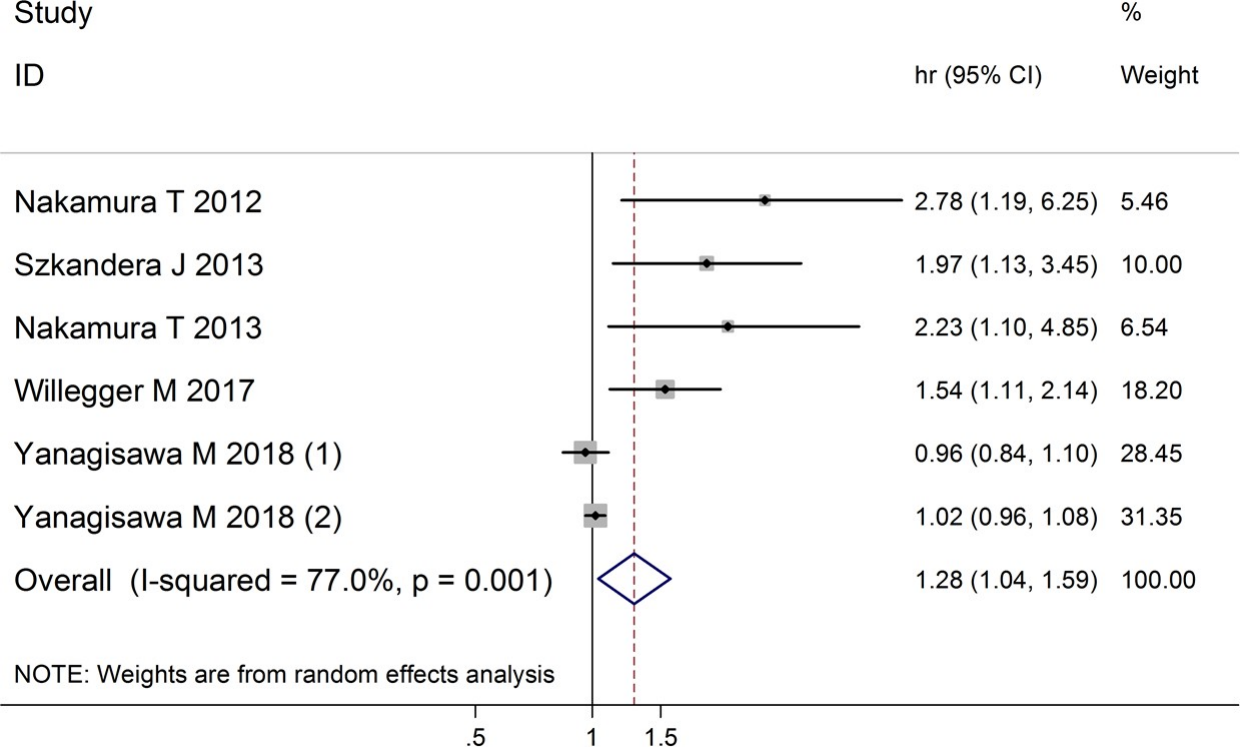
Forest plot showing the association between CRP levels and DFS/RFS of patients with soft tissue sarcoma. The summary HR and 95% CIs were shown.

Five articles including 6 independent patients groups reported the DFS/RFS data [14, 15, 17, 19, 22]. The random effects model revealed that higher CRP level was significantly correlated with poor DFS/RFS (HR = 1.28; 95% CI: 1.04–1.59; *p* = 0.022), but with significant heterogeneity (I^2^ = 77.0%, *p* = 0.001) (Fig 7).

#### Sources of heterogeneity

Meta-regression analysis (Table 4) were conducted to identify the sources of heterogeneity, and the results revealed that only the sample sizes (n > 100 or n < 100) were responsible for the heterogeneity (R^2^ = 100%, *p* = 0.012) with statistical significant. All of the survival analysis (local recurrence, distant recurrence or both local and distant recurrence), metastasis status (with or without metastasis at initial presentation), patients’ country regions (Europe, Asia or North America) and variable types (continuous or dichotomous) cannot explain the heterogeneity with statistical significant.

**Table 4:** Meta-regression analysis for the relationship between CRP levels and DFS/RFS.

Variables		*P* value	Tau^2^ value	Adj R^2^ value
**survival analysis**	local recurrence	reference	0	100.00%
	local or distant recurrence	0.576		
	distant recurrence	0.128		
**sample sizes**	n>100	reference	0	100.00%
	n<100	0.012		
**metastasis or not**	mixed	reference	0.113	5.13%
	without metastasis	0.508		
**country regions**	Asia	reference	0	100.00%
	Europe	0.352		
	North America	0.097		
**variable types**	continuous	reference	0.167	-39.33%
	dichotomous	0.909		

**Fig 8:**
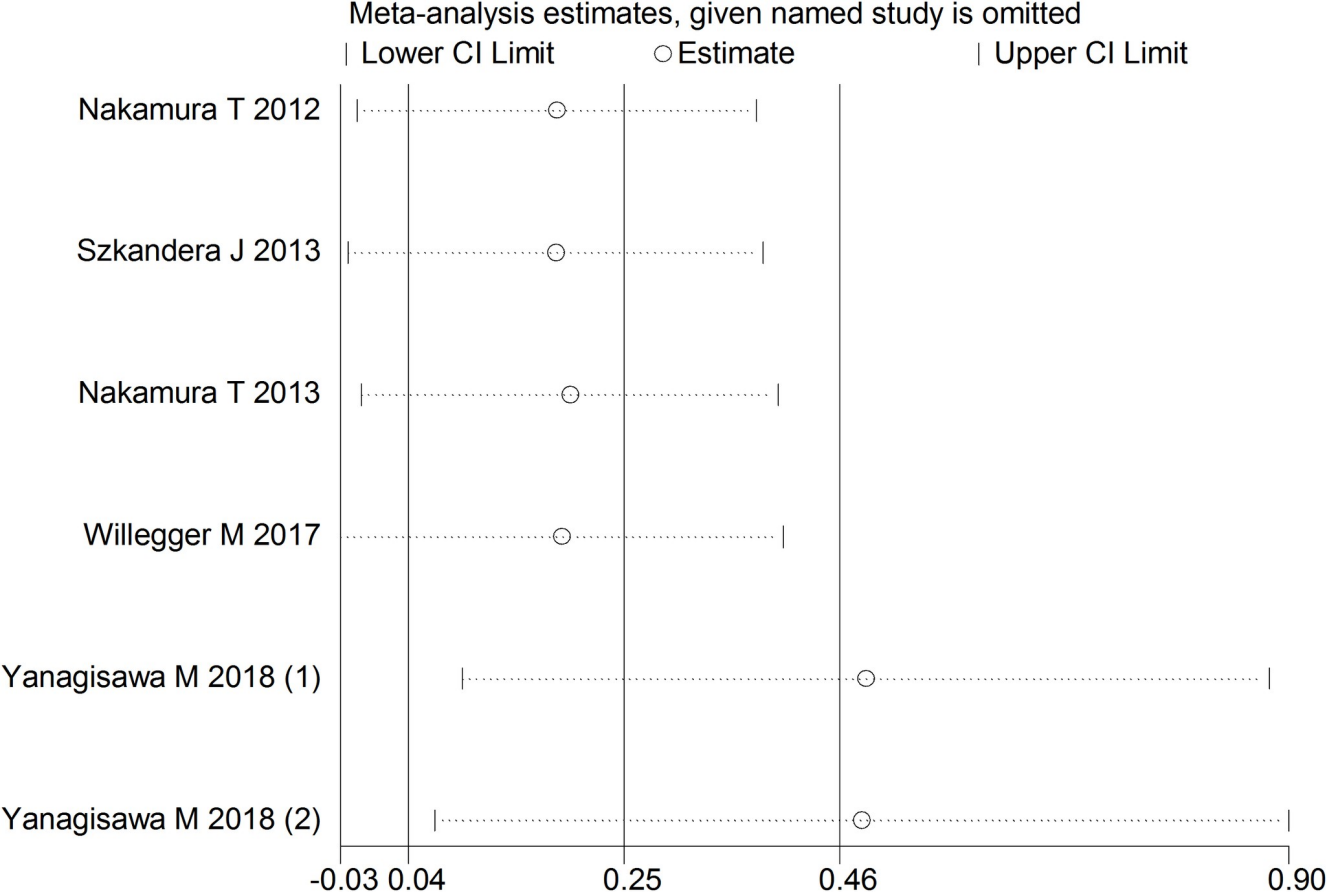
Sensitivity analysis for the relationship between CRP levels and DFS/RFS.

Sensitivity analysis was also conducted by excluding each one of the included studies. The results revealed that the study conducted by Yanagisawa M [22], which has two independent cohorts, had significant influence on the combined HR (Fig 8). This is consistent with the results of meta-regression analysis, as each of the two cohorts in this study have a small sample size (n = 49).

#### Subgroup analysis

**Fig 9:**
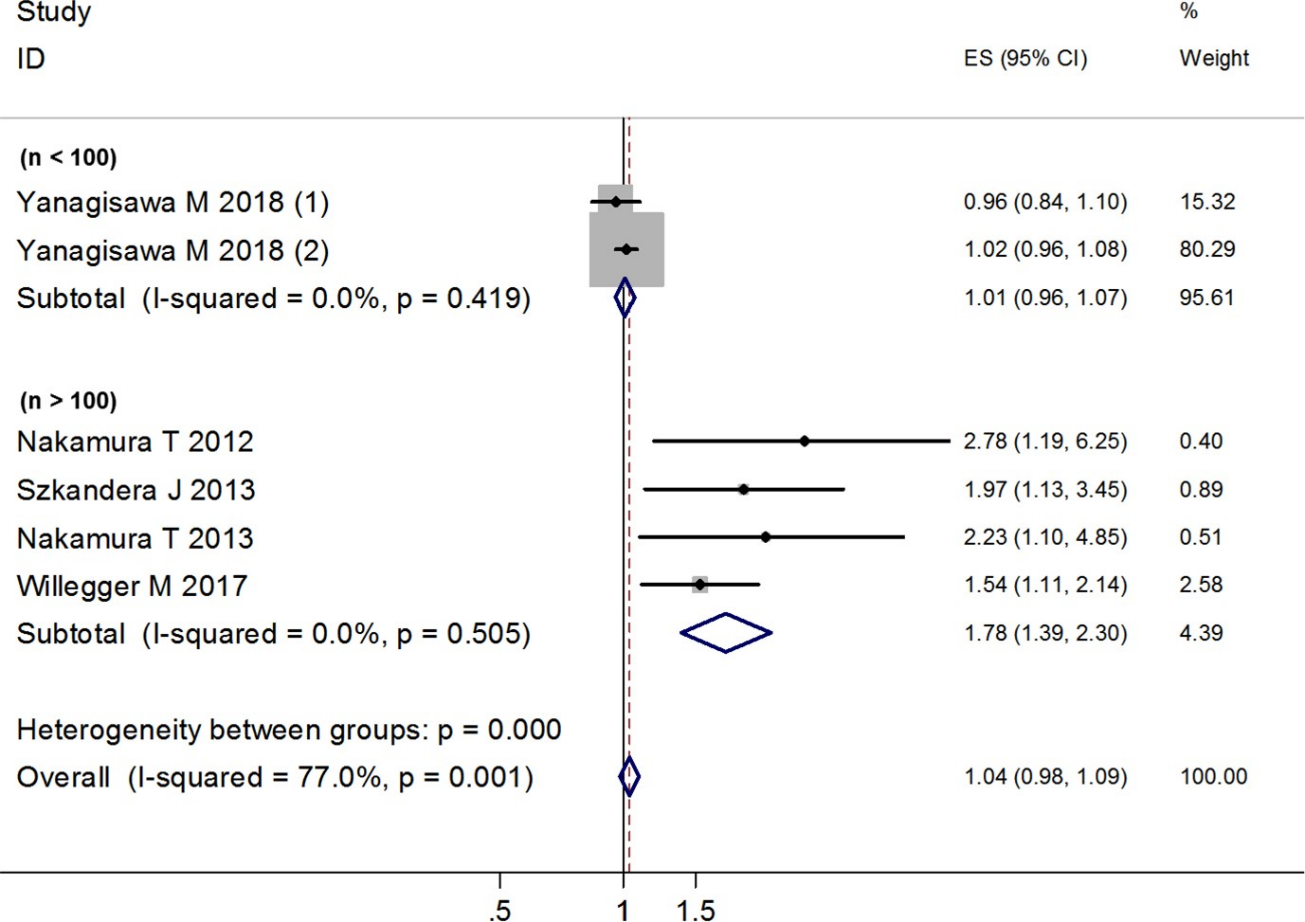
Forest plot of subgroup analysis for the relationship between CRP levels and DFS/RFS based on different sample sizes (n > 100 or n < 100).

Based on different sample sizes (n > 100 or n < 100), we divided the 6 studies into 2 groups: Group A, studies have more than 100 samples; group B, studies have less than 100 samples. There was no significant heterogeneity within each of the subgroup. For group A, the fixed effects model showed that higher CRP level was correlated with poor DFS/RFS (HR = 1.78; 95% CI: 1.39–2.30; *p* < 0.001); for group B, the fixed effects model showed that CRP level was not associated with DFS/RFS (HR = 1.01; 95% CI: 0.96–1.07; *p* = 0.714) (Fig 9).

#### Publication bias

**Fig 10:**
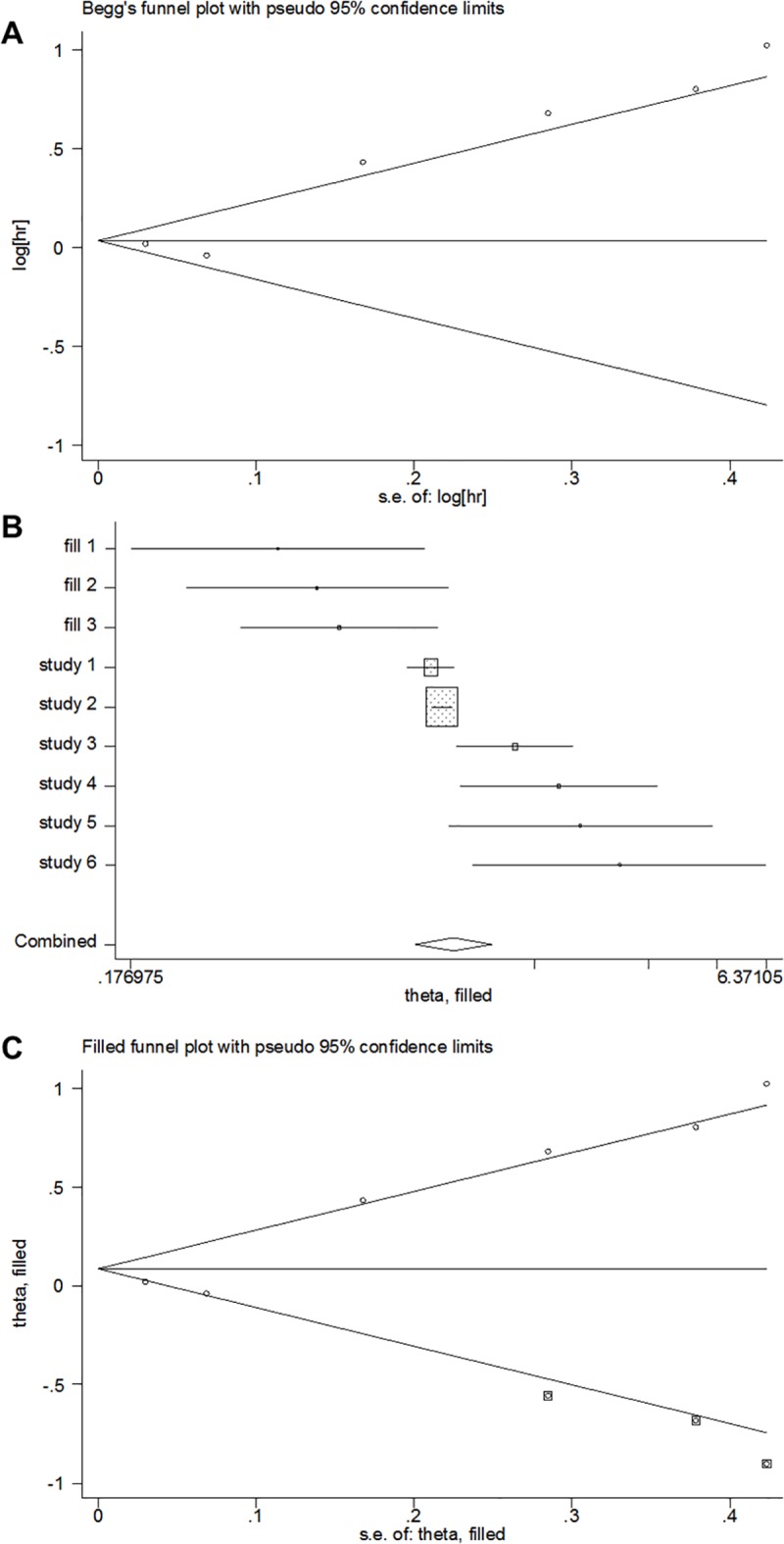
Analyses of publication bias for the relationship between CRP levels and DFS/RFS. (A) Funnel plot of all 6 studies. (B) Funnel plot after the trim and fill method was adopted. (C) Forest plot after the trim and fill method was adopted.

Among all the 6 included studies, the Begg’s test showed no evidence of significant publication bias (*p* = 0.452), but the Egger’s test showed evidence of significant publication bias (*p* = 0.019) (Fig 10A). After the trim and fill method was adopted, the results showed a lower HR value with no statistical significant (HR = 1.089; 95% CI: 0.877–1.353; *p* = 0.438; random effects) (Fig 10B and 10C).

## Discussion

Previously, a meta-analysis conducted by Li Y et al. [45] reported that higher level of pretreatment CRP level demonstrated a significantly higher risk of decreased recurrence and overall survival rates in both localized bone and soft tissue sarcomas together. But there were some deficiencies in their meta-analysis. Firstly, they did not separate STS from bone sarcoma, which represents a different group of sarcomas; Secondly, they combined both multivariate Cox hazard regression analysis data and univariate analysis data together, thus could not assess the independent role of CRP as a prognostic factor; Thirdly, they did not consider DSS as an endpoint, which might be more reliable than OS when it comes to sarcoma-caused death.

Our meta-analysis focused on the relationship between pretreatment serum CRP levels and DSS in STS patients. We combined only multivariable-adjusted hazard radios (HRs) to evaluate the independent prognostic role of CRP levels. The results showed that elevated CRP levels were independent prognostic factor for poor DSS, although there was significant heterogeneity among the 7 studies. Meta-regression analysis revealed that metastasis status and sample sizes were the main sources of heterogeneity. We then conducted subgroup analyses based on different metastasis status and different sample sizes respectively. The results also revealed that elevated CRP levels were significantly correlated with poor DSS in each of the subgroup, with no significant heterogeneity within each of the subgroup. The combined HR value of the subgroup which has more than 100 samples was higher than those of the subgroup which has less than 100 samples (HR = 2.40 vs HR = 1.11). Of course, the result of the subgroup with larger sample size was more convincing.

Sensitivity analysis revealed that the study conducted by Nakamura T et al. in 2017 had significant influence on the combined HR, which is the only one focus on metastatic STS patients and have the smallest sample size. After excluding this study, the heterogeneity among the remaining 6 studies disappeared, and the results showed that elevated CRP level was firmly correlated with poor DSS. Interestingly, this study also concluded that elevated CRP levels was associated with poor DSS, but with a relative lower HR value (HR = 1.11; 95% CI: 1.03–1.19; *p* = 0.007). Thus, it seems that the prognosis role of CRP levels is not as remarkable for metastatic STS patients as for non-metastatic STS patients. We presume that the relative lower HR value might be caused by relatively higher portion of patients with elevated CRP levels and relatively lower survival rate in the group of metastatic STS patients. In fact, in the study conducted by Nakamura T et al. in 2017, elevated pretreatment CRP levels were found in 43.5% of metastatic STS patients [21], higher than their previous studies where only 18 to 22% of the non-metastatic STS patients had elevated pretreatment CRP levels [15]. The 3-year DSS survival rate for metastatic STS patients with elevated and normal level of CRP were 15.6% and 47.1%, respectively [21]. But for non-metastatic STS patients, according to their previous study, the 3-year DSS survival rate were 49.4% in patients with elevated CRP level and 88.8% in patients with normal CRP level [15]. However, given that only one study with a small sample number have evaluated the prognostic role of CRP levels for metastatic STS patients, further large-scale study is warranted to draw a convincing conclusion.

Our present study also revealed that elevated CRP level was an independent prognostic factor for cofounder-adjusted DFS in the subgroup with studies have more than 100 samples (HR = 1.73; 95% CI: 1.33–2.27; P < 0.001), which meant that STS patients with elevated CRP level might be more likely to progress with local recurrence or distal metastasis. We presume that elevated serum CRP level might represent a more invasive propensity of STS. Although local condition could be controlled by surgical treatment and adjuvant radiotherapy, about 50% of patients with adequate local control develop distant metastases and ultimately die from their disease [2]. Recently, it has been shown that surgical margins are not predictive of local recurrence and survival in high grade myxofibrosarcoma [46, 47]. The inherent invasive characteristics of cancer might be more import when it comes to relapse or not, thus serum CRP level as well as other systemic inflammation markers might serve as an indicator for more intensive therapy. Indeed, researchers also found that combination use of different serum inflammation markers, such as Glasgow Prognostic Score (GPS), neutrophil/lymphocyte ratio (NLR) and C-reactive protein/Albumin Ratio (CAR), could predict prognosis of sarcoma patients [18, 24, 48, 49], highlighting the import of sarcoma-associated inflammation responses.

It should be noticed that the study conducted by Yanagisawa M [22] revealed pretreatment CRP level not predictive of worse DRFS, neither for the group received upfront surgery (HR = 1.02; 95% CI: 0.96–1.08; P = 0.49) nor for the group received neoadjuvant radiotherapy (HR = 0.96; 95% CI: 0.84–1.10; P = 0.54). This discrepancy could be explained by the following reasons. Firstly, either the upfront surgery cohort (n = 49) or the neoadjuvant radiotherapy cohort (n = 49) in this study has a very small sample size, while the other four included studies all have a sample size more 100 patients. There is no doubt that the results of the studies with larger sample size are more convincing. Secondly, this study only investigate distant metastasis, while 1 of the other 4 studies investigated local recurrence, and the other 3 investigated either local recurrence or distant metastasis. Elevated CRP levels might have different prediction role for local recurrence and distant metastasis, and further researches are warranted.

Interestingly, although Yanagisawa M [22] did not found pretreatment CRP level predictive of worse DRFS in either upfront surgery cohort or neoadjuvant radiotherapy cohort, the author observed that pretreatment CRP level was predictive of worse OS (HR = 1.16; 95% CI: 1.05–1.29; P = 0.003) in upfront surgery cohort, but not in neoadjuvant radiotherapy cohort. As the author pointed out in the paper, the results mentioned above suggest that neoadjuvant radiotherapy may impact the inflammatory milieu of the tumor microenvironments in a different manner than upfront surgery and thereby alter the interaction of these biomarkers with outcome [22]. There are some studies suggested that complex inflammatory and cell-signaling cascades secondary to radiotherapy might mediate a strong immune response which can counteract or even overcome the local immunosuppression of the tumor microenvironments [50]. The utility of CRP as predictors of worse clinical outcome may not apply in patients received neoadjuvant RT, but patients with elevated CRP at diagnosis may be good candidates for neoadjuvant RT.

Immune check point antibody inhibitors, such as anti-PD-1/PD-L1, are rapidly becoming a highly promising cancer therapeutic approach that yields remarkable antitumor responses with limited side effect [51]. Sarcoma has not traditionally been considered an immunogenic tumor, however, several studies showed PD-L1 to be expressed in up to 30–40% of certain sarcoma subtypes [52, 53]. A recent study also found that Pembrolizumab (an anti-PD-1 antibody) monotherapy was associated with clinically meaningful and sustained objective response in seven (18%) of 40 patients with soft-tissue sarcoma [53]. More interestingly, a study found that high level of preoperative serum CRP was significantly associated with PD-L1 positivity in 508 patients with non-small cell lung cancer [13]. Elevated CRP levels might represent high level of PD-L1 expression in malignancies including soft tissue sarcomas, thus CRP might have a role to serve as an indicator for immune checkpoint blockade therapy with anti-PD-1 antibodies. Further investigation is required to confirm the correlation of CRP levels and PD-L1 expression levels in soft tissue sarcomas.

There were some limitations in this meta-analysis. Firstly, only a small number of studies were included in this meta-analysis, and most of the studies were retrospective researches. Secondly, there are some inherent heterogeneity among those included studies, such as different patient’s source, different cut-off methods and cut-off value, different sample sizes, and different variables for multivariate analysis. We did Meta-regression analysis, sensitivity analysis and subgroup analysis to find the source of heterogeneity and assess their influence on the results. Thirdly, most of the included studies did not take systemic inflammatory conditions into consideration. We chose the DSS and DFS as the primary outcome to minimize the influence of systemic inflammatory conditions on the survival of STS patients. Fourthly, STS represents a heterogeneous group of tumors including multiple subtypes probably with different status of inflammatory response. Moreover, anti-PD-1 therapy using pembrolizumab observed different objective response rate for different subtypes of STS: 40% for undifferentiated pleomorphic sarcoma, 20% for liposarcoma, 10% for synovial sarcoma, and none for leiomyosarcoma [53]. It would be better to analyze STS based on different histology subtypes. However, in the present meta-analysis, except for the study conducted by Panotopoulos J et al. [44] investigating only liposarcoma, all of the other included studies involved multiple subtypes of STS, as is shown in Table 1. Thus, we could not subdivide groups by histology in the meta-analysis. It also has to be noted that liposarcoma comprise several subtypes such as well-differentiated liposarcoma, dedifferentiated liposarcoma, myxoid liposarcoma, round cell liposarcoma and pleomorphic liposarcoma [54]. Well-differentiated liposarcoma (WDL) is rarely, if at all, fatal, and now is commonly referred to as atypical lipomatous tumor [55]. WDL does not have malignant potential itself. It has a propensity for local recurrence but lacks metastatic capacity, and has a rare risk of de-differentiating into a sarcomatous condition such as dedifferentiated liposarcoma [54, 55]. However, in the present meta-analysis, one study [14] includes WDL and dermatofibrosarcoma protuberance (DFSP). Similar to WDL, DFSP may also recur but has a very low risk of metastasis [56]. Many of the other included studies list liposarcoma too, but it is unclear how many of these included are WDL. We recommend further researches should focus on different histologic subtypes of STS specifically.

For the clinical use of CRP as prognostic indicator for STS patents, the most imperative thing is to ascertain the optimal cut-off value. However, since the included studies adopted several different cut-off values from various types of method, we cannot draw a conclusion for the optimal cut-off value of CRP in the present study. Three of the included studies chose the cut-off value according to clinical routine (2 use 0.3 mg/dL, 1 use 1 mg/dL) [14, 15, 21]; One use 0.3 mg/dL as cut-off value by reference other researches [20]; One use median value (0.5 mg/dL) as cut-off value [22]; Two use ROC curve to ascertain the optimal cut-off value (0.2 mg/dL and 0.69 mg/dL, respectively) [17, 18]. CRP is a mature clinical indicator for inflammation response, and elevated CRP levels indicate the evidence of inflammation response. It seems to be plausible and convenient to use the clinical routine (higher than the normal CRP levels) as the cut-off value to predict survival or indicate more aggressive therapy for STS patients. However, the normal CRP level varies among different medical institutions due to different testing methods or calibrations. The optimal cut-off value of CRP for risk stratification or as a base for more aggressive therapy is still to be determined. More data and researches are still needed.

In conclusion, our meta-analysis suggests that elevated pretreatment serum CRP level could serve as independent prognostic indicator for DSS and DFS/RFS in STS patents. Elevated CRP level as well as other systemic inflammation markers might represent inherent aggressive characteristic of STS, and might serve as indicators for more intensive therapy such as immune checkpoint blockade therapy using anti-PD-1 antibodies. Well-designed large-scale prospective clinical researches are warranted. More fundamental researches are also required to elucidate the hidden mechanism of sarcoma-associated inflammation responses.

## Supporting information

S1 Checklist PRISMA checklist for meta-analysis of the prognostic value of C-reactive protein in patients with soft tissue sarcoma. (DOC)
